# Real-Time Imaging of Resident T Cells in Human Lung and Ovarian Carcinomas Reveals How Different Tumor Microenvironments Control T Lymphocyte Migration

**DOI:** 10.3389/fimmu.2015.00500

**Published:** 2015-10-12

**Authors:** Houcine Bougherara, Audrey Mansuet-Lupo, Marco Alifano, Charlotte Ngô, Diane Damotte, Marie-Aude Le Frère-Belda, Emmanuel Donnadieu, Elisa Peranzoni

**Affiliations:** ^1^INSERM U1016, Institut Cochin, Paris, France; ^2^CNRS, UMR8104, Paris, France; ^3^Université Paris Descartes, Sorbonne Paris Cité, Paris, France; ^4^Department of Pathology, Paris Centre University Hospitals, Assistance Publique-Hôpitaux de Paris, Paris, France; ^5^Cancer and Immune Escape, Cordeliers Research Center, INSERM U1138, Paris, France; ^6^University Pierre and Marie Curie, Paris, France; ^7^Department of Thoracic Surgery, Paris Centre University Hospitals, Assistance Publique-Hôpitaux de Paris, Paris, France; ^8^Department of Gynaecological and Oncological Surgery, Hôpital Européen Georges Pompidou, Université Paris Descartes, Assistance Publique-Hôpitaux de Paris, Paris, France; ^9^Department of Pathology, Hôpital Européen Georges Pompidou, Université Paris Descartes, Assistance Publique-Hôpitaux de Paris, Paris, France

**Keywords:** T cells, human cancer, stroma, migration, imaging, extracellular matrix, lung cancer, ovarian cancer

## Abstract

T cells play a key role in the battle against cancer. To perform their antitumor activities, T cells need to adequately respond to tumor antigens by establishing contacts with either malignant cells or antigen-presenting cells. These latter functions rely on a series of migratory steps that go from entry of T cells into the tumor followed by their locomotion in the tumor stroma. Our knowledge of how T cells migrate within tumors mainly comes from experiments performed in mouse models. Whereas such systems have greatly advanced our understanding, they do not always faithfully recapitulate the disease observed in cancer patients. We previously described a technique based on tissue slices that enables to track with real-time imaging microscopy the motile behavior of fluorescent T cells plated onto fresh sections of human lung tumors. We have now refined this approach to monitor the locomotion of resident tumor-infiltrating CD8 T cells labeled with fluorescently coupled antibodies. Using this approach, our findings reveal that CD8 T cells accumulate in the stroma of ovarian and lung carcinomas but move slowly in this compartment. Conversely, even though less populated, tumors islets were found to be zones of faster migration for resident CD8 T cells. We also confirm the key role played by collagen fibers, which, by their orientation, spacing and density, control the distribution and migration of resident CD8 T cells within the tumor stroma. We have subsequently demonstrated that, under some physical tissue constraints, CD8 T cells exhibited a mode of migration characterized by alternate forward and backward movements. In sum, using an *ex vivo* assay to track CD8 T cells in fresh human tumor tissues, we have identified the extracellular matrix as a major stromal component in influencing T cell migration, thereby impacting the control of tumor growth. This approach will aid in the development and testing of novel immunotherapy strategies to promote T cell migration in tumors.

## Introduction

The importance of T lymphocytes in antitumor immunity is by now well established. In recent years, the extensive knowledge in this field has finally found a promising clinical translation with the adoptive cell transfer (ACT) of tumor antigen-specific T cells and the development of immune checkpoint inhibitors, as anti-PD-1 and anti-CTLA-4, which have proven to be effective in a number of cancer patients (1).

Current immunotherapies mainly aim at increasing tumor-specific T cell number or boost their cytotoxicity. However, these treatments do not benefit all cancer patients, revealing the presence of other obstacles that need to be identified. These can concern defects in the ability of T cells to respond to tumor antigens and a failure of lymphocytes to interact with malignant cells and/or with antigen-presenting cells. In particular, in order to exert a direct antitumor cytotoxic activity, T lymphocytes need not only to be recruited to the tumor site but should also make a direct physical contact with tumor cells (2) and cooperate with other immune partners. This T cell–tumor cell interaction is the end result of a number of migratory steps, that start when lymphocytes cross the venular wall to enter into the tumor, then migrate within the stroma, a complex microenvironment composed of non-cancer cells along with the extracellular matrix (ECM), and eventually make contact with malignant cells. Whereas a lot of attention has been paid to the extravasation step, its defects, and how to overcome them (3), much less is known about the intra-tumoral motile behavior of T cells, especially in human malignant tissues.

In mouse models, the dynamics of T cells in the tumor microenvironment have been followed in real time by intravital microscopy. Most studies have explored the behavior of adoptively transferred or endogenous fluorescent T cells in the early rejection phase, both by two-photon (4–9) and confocal (10) imaging. These experiments have shed light on how T cells migrate within tumors and interact with tumor cells and antigen-presenting cells, but they have been primarily performed with transplantable, antigen-specific tumor models, thus not fully recapitulating the situation in human cancer.

Up to the present, all the data available for T cells in human tumors mainly come from immunostainings performed on fixed tissues. These studies have revealed that growing human tumor T cells are usually much more abundant in the stroma compared to tumor islets, highlighting the presence of obstacles that limit T cells from infiltrating tumor cell regions (2, 11). Conversely, the presence of CD3^+^ and/or CD8^+^ T cells in tumor nests relative to stroma is correlated with a better prognosis and better response to therapy in several human cancers (12–17). Moreover, the notion that T cells are hindered in their trafficking into and within tumors has been supported in mice by the synergy between therapeutic immunotherapies and approaches that target different components of the stroma and enhance T cell infiltration into tumor nests (18–21).

To gain insight into lymphocyte dynamics in a human 3D tumor microenvironment, we have recently developed a method to monitor the motility of T cells plated on top of fresh tumor tissue slices (11, 22). In such conditions, exogenously added fluorescently labeled lymphocytes closely mimic the spatial organization of their endogenous counterpart and have allowed us to point out some of the obstacles that limit T cells in their capacity to get in close contact with tumor cells. In particular, our data reveal that the density and orientation of collagen fibers of the ECM control the distribution and migration of T cells within tumors and their ability to infiltrate tumor islets.

However, this approach has some limitations. It relies on T cell purification, which is not always possible for all the samples, due to the reduced size or the scarcity of T cells of some biopsies. In addition, we do not know whether the behavior of purified or peripheral blood activated T cells truly mimics the behavior of resident intratumoral T lymphocytes. We have thus refined our slice assay by using a direct staining of endogenous cells with fluorescent antibodies in fresh tumor slices. This allowed us to track for the first time the dynamics of resident CD8 T cells in human tumors with a combination of confocal and multi-photon microscopy. This novel technique also permits the investigation of real-time interactions between lymphocytes and other cell types, such as tumor or stromal cells, as well as ECM components.

We have thus confirmed our previous results by observing the same key role of the ECM in controlling endogenous CD8 T cell behavior in human lung cancer, and we have extended these findings to another human tumor, the ovarian epithelial carcinoma. Our data unveil different CD8 T cell dynamics on the basis of their localization (stromal vs. intratumoral) and a new mode of T cell migration, characterized by alternate forward and backward movements, determined by the physical constraints of the ECM.

## Materials and Methods

### Human tumors

Fresh lung tumors were obtained from anonymized patients diagnosed with clinical stage I–III non-small cell lung cancer and who underwent radical lobectomy or pneumonectomy. No chemotherapy or radiotherapy was administered before the operation. Non-fixed fresh tumors obtained after the resection were rapidly transported to the laboratory in ice-cold RPMI 1640. A total of 19 lung tumors were used for this study, both adenocarcinomas and squamous cell carcinomas, among which 12 were discarded due to a scarcity of T cells in the tumor tissue, to very high level of autofluorescence of fibers precluding imaging of T cells, to an unclear distinction between tumor cell regions and stroma and to complete immobility of CD8 T cells in the whole tissue. These clear qualitative differences between tumors were noticed after visual inspection of several microscopic fields or time-lapse imaging of immunostained tumor slices. Fresh ovarian tumors were obtained from anonymized patients diagnosed with clinical stage III–IV epithelial ovarian cancer and who underwent exploratory laparotomy or complete surgery. Non-fixed fresh tumors obtained after the resection were rapidly transported to the laboratory in ice-cold RPMI 1640. A total of 15 epithelial ovarian cancers were used for this study, both endometrial and serous adenocarcinomas, among which 6 were discarded due to a scarcity of T cells in the tumor tissue, to an unclear distinction between tumor cell regions and stroma and to complete immobility of CD8 T cells in the whole tissue.

Human tumors were obtained with the agreement of the French ethical committee, and by the Assistance Publique-Hôpitaux de Paris (AP-HP), in application with the article L.1121-1 of French law. A written informed consent was obtained from the patients prior to inclusion in the study.

### Tumor slices and immunostaining

Experiments were performed with tumor specimens obtained 2–4 h after tumor resection. A minority of biopsies was kept 12–24 h at 4°C before processing for imaging and similar results were obtained with these samples in terms of T cell motility. Tumor slices were prepared as described in Refs. (11, 22). In brief, samples were embedded in 5% low-gelling-temperature agarose (type VII-A, Sigma-Aldrich) prepared in PBS. 350 μm slices were cut with a vibratome (VT 1000S, Leica) in a bath of ice-cold PBS. Slices were transferred to 0.4-μm organotypic culture inserts (Millicell, Millipore) in 35-mm Petri dishes containing 1 ml RPMI 1640 in an incubator at 37°C/5% CO_2_.

Live vibratome sections were stained for 15 min at 37°C with the following antibodies: PE-conjugated anti-CD8 (clone SK1, purchased from BD Biosciences), FITC-conjugated anti-EpCAM (clone HEA-125, purchased from Miltenyi) and washed thereafter. In some experiments, slices were stained with AlexaFluor647-conjugated anti-CD8 Fab fragment (R&D Biotech). All antibodies were diluted in RPMI and used at a concentration of 10 μg/ml. To concentrate the antibodies on the tissue, a stainless steel ring was placed to the agarose surrounding the slice.

### Time-lapse imaging

T cells were imaged with a DM500B upright microscope equipped with a SP5 confocal head (Leica) and a 37°C thermostated chamber. For dynamic imaging, tumor slices were secured with a stainless steel slice anchor (Warner Instruments) and perfused at a rate of 1 ml/min with a solution of RPMI without phenol red, bubbled with 95% O_2_ and 5% CO_2_. Ten minutes later, images from a first microscopic field were acquired with a 25× water immersion objective (Olympus, 20×/0.95 NA). For four-dimensional analysis of cell migration, stacks of 6–10 sections (*z* step = 5–7 μm) were acquired every 30 s for 20–40 min, at depths up to 80 μm. Regions were selected for imaging when tumor parenchyma, stroma and T cells were simultaneously present in the same microscopic field. For most of the tumors included in the study, between 2 and 4 microscopic fields were selected for time-lapse experiments.

For two-photon imaging, excitation was provided by a Chameleon Ultra Ti:Sapphire laser (Coherent). Emitted fluorescence was detected through 405/15 (SHG), 525/50 (Alexa-488) and 610/50 (PE) non-descanned detectors (NDD). For confocal imaging, excitation was provided by an Ar laser (488 nm excitation) and a HeNe laser (633 nm excitation) and emitted fluorescence was detected in the following PMT spectra ranges: 500–560 nm (FITC, alexa-488), 560–630 nm (PE) and 640–750 nm (APC, alexa-647).

### Data analysis

Image analysis was performed at the Cochin Imaging Facility (Institut Cochin, Paris). A 3D image analysis was performed on *x*, *y*, and *z* planes using Imaris 7.4 (Bitplane AG). First, superficial planes from top of the slice to 15 μm in depth were removed to exclude T cells located near the cut surface. Cellular motility parameters were then calculated using Imaris. Tracks >10% of the total recording time were included in the analysis. The straightness value was calculated as the ratio of the distance from origin to the total distance traveled. To reveal the relationship between CD8 T cell motility and the tumor structure (tumor islets and collagen network), confocal time-lapse images of T cells were superimposed onto the corresponding SHG and EpCAM images. CD8 T cells localized in the stroma were distinguished from those infiltrated in tumor cell nests by looking at individual planes along the *z* axis. Videos and images were made by compressing the *z* information into a single image using Imaris. When a drift in the *x*, *y* dimension was noticed, it was corrected using the “Correct 3D Drift” plug-in in ImageJ. For the automated detection of resident CD8 T cells in different tumor areas (stroma, tumor islets, loose, and dense collagen regions identified by visual inspection of SHG images), we used the ImageJ software. First, fluorescent images were thresholded and converted to binary images. Angles between the cell trajectory vectors, which are the connecting lines between starting points and end points of each track, and tumor-stroma boundaries were calculated using Image J software. Only the cells positioned within a maximum distance of 100 μm from the tumor-stroma interfaces were included in further analysis. Distances between collagen fibers were determined by using the “point to point distance measurement” function of Imaris.

### Statistical analysis

We first used a Kolmogorov–Smirnov normality test (one sample test) to determine whether data values distributed normally. When values were not normally distributed, an unpaired two-tailed non-parametric Mann–Whitney test was performed to determine statistical significance. When values followed a Gaussian distribution, an unpaired *t*-test was performed to determine statistical significance. All these statistical analysis were performed with Prism 6 (GraphPad). *P < 0.05; **P < 0.01; ***P < 0.001.

## Results

### Tracking resident CD8 T cell motility in human tumors

**Figure 1:**
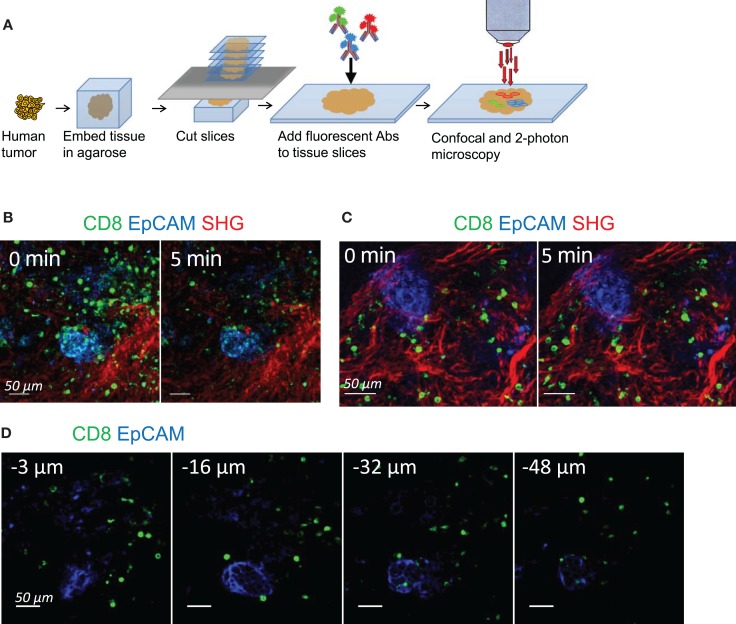
**Tracking resident CD8 T cell motility in human tumors**. **(A)** Experimental setup. Tissue slices were prepared from human lung and ovarian tumors by means of a vibratome. Slices were then stained with conjugated antibodies and imaged through confocal and two-photon microscopy. **(B)** Snapshots of two-photon images of an ovarian tumor slice stained for CD8 (green) and EpCAM (blue) to identify tumor cell regions. SHG (red) was used to visualize the collagen network. See also Movie S1 in Supplementary Material. **(C)** Snapshots of confocal images of an ovarian tumor slice stained for CD8 (green) and EpCAM (blue) to identify tumor cell regions. Second-harmonic generation images (red) captured with a two-photon microscope were superimposed to the confocal images. See also Movie S2 in Supplementary Material. **(D)** Snapshots of confocal images of an ovarian tumor slice stained with the indicated antibodies and captured at different depths from the cut surface. See also Movies S3 and S4 in Supplementary Material.

In order to track the dynamic behavior of resident CD8 T cells in human ovarian and lung tumors, we have modified our previously described slice preparation (11). A fragment of tumor rapidly obtained after surgical resection was embedded in agarose and sliced into 350 μm-thick sections with a vibratome (Figure 1A). Unfixed slices were then labeled with fluorescently coupled anti-CD8 and anti-EpCAM antibodies to visualize CD8 T cells and tumor epithelial cells, respectively. The fibrillar collagen was assessed without exogenous staining by using second-harmonic generation (SHG).

**Figure 2:**
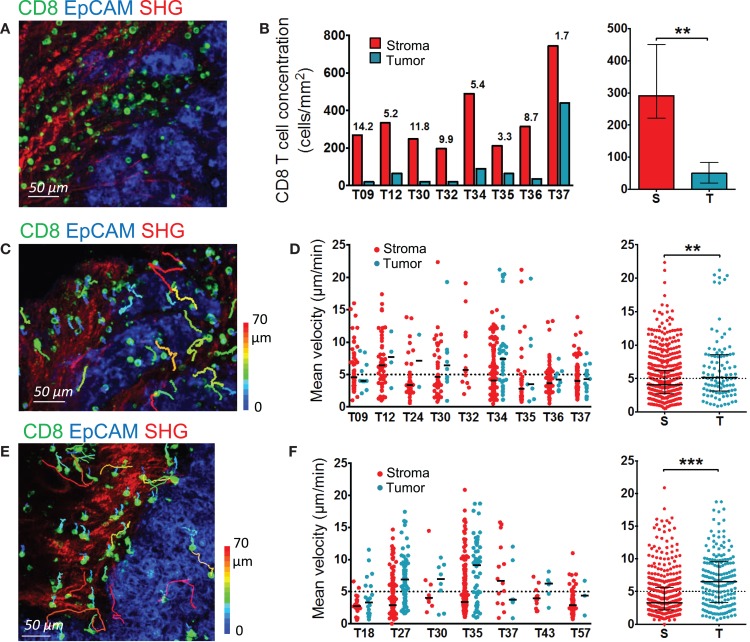
**Distribution and migration of resident CD8 T cells in human ovarian and lung tumors**. **(A,B)** Distribution of resident CD8 T cells in human ovarian tumors. **(A)** Representative image of a human ovarian tumor slice stained for EpCAM (blue) and CD8 (green). The SHG signal is in red. **(B)** Concentration of resident CD8 T cells in the stromal and tumor cell regions of human ovarian carcinomas. Left panel: CD8 density in stromal (red) and tumor cell (blue) regions for individual tumors. Numbers indicate the fold increase of CD8 T cells in stromal regions over tumor islets. Right panel: pooled values, median ± interquartile range. **(C,D)** Motility of resident CD8 T cells in human ovarian tumors. **(C)** Trajectories of individual resident CD8 T cells in the stromal (red) and tumor cell (blue) regions of a human ovarian carcinoma slice. See also Movie S5 in Supplementary Material. The slice was stained with the indicated antibodies and imaged for 30 min with a confocal microscope. The SHG signal was recorded at the end with a two-photon microscope. Tracks are color-coded according to CD8 T cell displacement length. **(D)** Left panel: medians of mean velocities of resident CD8 T cells in the stromal and tumor cell regions of individual human ovarian carcinomas. Right panel: pooled values, medians ± interquartile. **(E,F)** Motility of resident CD8 T cells in human lung tumors. **(E)** Trajectories of individual resident CD8 T cells in the stromal (red) and tumor cell (blue) regions of a human lung carcinoma slice. The slice was stained with the indicated antibodies and imaged for 20 min with a confocal microscope. The SHG signal was recorded at the end with a two-photon microscope. Tracks are color-coded according to CD8 T cell displacement length. **(F)** Left panel: medians of mean velocities of resident CD8 T cells in the stromal and tumor cell regions of individual human lung carcinomas. Right panel: pooled values, medians ± interquartile. Mann–Whitney test, *P < 0.05; **P < 0.01; ***P < 0.001.

Time-lapse experiments were first performed with a two-photon microscope. Stacks of images from the cut surface to 80 μm in depth were captured sequentially every 15–30 s for 20 min. A representative example of CD8 T cell distribution and migration in ovarian tumor slices is shown in Figure 1B and Movie S1 in Supplementary Material. The structure is typical of a carcinoma, with well-delineated and compact tumor islets positive for EpCAM, surrounded by a stroma enriched in collagen. Resident CD8 T cells were preferentially found in the stroma, with some of them being actively motile (see also Figure 2). However, with two-photon imaging a pronounced photobleaching occurred and after 5 min of recording the fluorescence signal of CD8 T cells was largely decreased. As this rather fast bleaching of the organic dyes complicated the analysis of T cell dynamics, we decided to switch to confocal microscopy. Movie S2 in Supplementary Material and Figure 1C show that the photobleaching was considerably reduced using this approach. In this setting, stacks of EpCAM and CD8 images from the cut surface to 80 μm in depth were recorded over 20–30 min with the confocal mode. Then, at the end of the recording, one stack of SHG images was captured with the two-photon mode and superimposed to the EpCAM and CD8 stack of images.

Given the size of antibodies, it was important to make sure that they could efficiently penetrate the tumor tissue. Figure 1D shows confocal planes of a microscopic field captured at 3, 16, 32, and 48 μm below the cut surface of a slice labeled for CD8 and EpCAM. Even if the fluorescence signal of anti-CD8 antibody was stronger in the superficial region of the slice (3 μm), CD8 T cells were still easily detected deeper within the tissue, at depths up to 50 μm. In line with our previous study in murine lymph nodes (23), we noted that a large proportion of CD8 T cells in the superficial regions of the slice was round and stationary (Movie S3 in Supplementary Material), likely reflecting a tissue damage associated with the slicing procedure. Accordingly, a large fraction of cells at the cut surface of the slice was labeled with DAPI, a nuclear dye that only penetrates cells whose plasma membrane is damaged (data not shown). However, below 15 μm of depth, virtually all motile and sessile CD8 T cells were viable (Movie S4 in Supplementary Material and data not shown).

These results demonstrate that antibody labeling of unfixed human tumor slices combined with confocal and two-photon microscopy enabled to dynamically monitor resident CD8 T cells in a native three-dimensional tumor environment, with minimal photobleaching and without the need for T cell purification, staining and plating. This setting, which has the advantage to examine T cells in human tumors under physiological conditions, was subsequently used to perform an in-depth analysis of CD8 T cell distribution and migration in ovarian and lung carcinomas.

### Resident CD8 T cell distribution and migration in human ovarian and lung tumors

Our previous study conducted on non-small cell lung tumors revealed the preferential accumulation of endogenous and plated T cells in the stroma rather than in the tumor epithelial compartment (11). Here, we analyzed the localization and motile behavior of endogenous CD8 T cells in different regions of ovarian carcinomas.

In ovarian carcinomas, CD8 T cells were preferentially enriched in the stroma and rarely found in EpCAM^+^ tumor cell regions (Figure 2A), as previously observed in lung tumors. Analysis performed in slices from nine different human ovarian tumors showed on average five times more CD8 T cells in the stroma compared to tumor islets (Figure 2B). Notably, some large variations from one tumor to another were observed, with some tumors showing tumor islets nearly devoid of CD8 T cells.

We then determined the motile behavior of resident CD8 T cells in the stroma and tumor cell regions of slices from ovarian carcinomas. The motility of CD8 T cells localized in the stroma was rather low, with a median of mean velocity of 4.04 μm/min (Figures 2C,D and Movie S5 in Supplementary Material). We also quantified the percentage of migrating cells, defined as cells with a mean velocity higher than 5 μm/min during a 20-min recording. Data from nine different ovarian tumors indicated that 37% of CD8 T cells were motile. Large variations were noted from one tumor to the other, with proportion of migrating CD8 T cells ranging from 20 to 60%. Of interest, the few CD8 T cells located in tumor islets migrated more rapidly than those found in the stroma with a median of mean velocity of 5.16 μm/min (Figure 2D and Movie S6 in Supplementary Material). We confirmed these findings in lung tumors (Figures 2E,F). In the stroma of lung tumors, the median of mean velocity was 3.27 μm/min, whereas in tumor islets the velocity was two-fold higher (6.53 μm/min). The difference between these velocities is significant (*P* < 0.0001). Highly motile CD8 T cells were deeply infiltrated into tumor islets, as shown in Movies S7 and S8 in Supplementary Material that represent T cell dynamics in individual z planes.

Since full-sized antibodies have, in principle, the potential to bind to Fc receptors and to transmit signals that can significantly impact T cell motility, we also performed experiments with a fluorescently labeled Fab fragment directed against CD8 that confirmed the conclusions drawn with full-sized antibodies. Thus, as shown in Movie S9 in Supplementary Material, most CD8 T cells in the stroma of an ovarian tumor slice displayed low or no motility, with only rare cells migrating.

These results indicate that in ovarian and lung carcinoma, resident CD8 T cells preferentially accumulate in the stroma, and move slowly in this region. Conversely, even though less populated, tumors islets were found to be zones of faster migration for resident CD8 T cells.

### Collagen fibers control the distribution and migration of CD8 T cells in the stroma of human ovarian carcinoma

**Figure 3:**
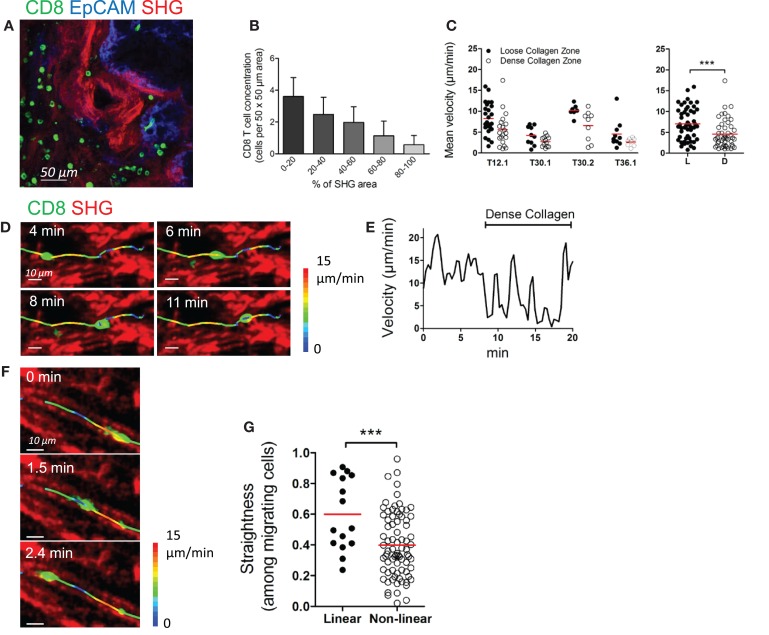
**Collagen fibers control the distribution and migration of CD8 T cells in the stroma of human ovarian carcinoma**. **(A)** Human ovarian tumor slice stained for CD8 (green) and EpCAM (blue), showing accumulation of resident CD8 T cells in collagen-loose regions. Collagen (red) has been imaged through SHG. **(B)** Automatically counted number of resident CD8 T cells in different 50 μm × 50 μm stromal regions classified according to the surface (%) occupied by collagen detected through SHG. Results were obtained on slices from four different ovarian tumors. **(C)** Left panel: mean velocities of resident CD8 T cells in loose and dense collagen areas of individual human ovarian carcinomas. Right panel: pooled values, mean ± SD. **(D–G)** Motile behavior of resident CD8 T cells relative to collagen fibers. **(D)** Snapshots are shown at various time intervals for the same CD8 T cell. The trajectory of this CD8 T cell was superimposed over SHG images. The track is color-coded according to the instantaneous velocity. See also Movie S11 in Supplementary Material. **(E)** Velocity of the CD8 T cell represented in **(D)**. **(F)** Snapshots are shown at various time intervals for the same CD8 T cell located in a linear collagen region. The trajectory of the CD8 T cell was superimposed over SHG images. The track is color-coded according to the instantaneous velocity. See also Movie S12 in Supplementary Material. **(G)** The graph shows the straightness index of individual tracks of CD8 T cells in linear and non-linear collagen fiber regions. Straightness indices close to 1 correspond to linear trajectories. Values are compiled from four different ovarian tumors. Mann–Whitney test, *P < 0.05; **P < 0.01; ***P < 0.001.

The motility of CD8 T cells in the stroma does not only depend on T cell properties and response to chemokines, but also on external constraints that may limit interstitial lymphocyte migration. We have previously demonstrated the importance of the structure of the stroma in the distribution and migration of plated T cells in human lung tumors (11). Here, we examined the organization of the collagen network in ovarian tumors and their influence on CD8 T cell distribution and migration. Collagen-rich regions were frequently observed in the stroma of ovarian carcinomas (Figure 3A). Our data indicate that dense regions contained less CD8 T cells than loose-collagen areas, as the number of CD8 T cells in defined stromal areas was inversely correlated with the amount of collagen in the same areas (Figure 3B). Likewise, the motility of CD8 T cells in dense collagen regions of the stroma was decreased compared to porous collagen regions, where fast moving cells were frequently observed (Figure 3C and Movie S10 in Supplementary Material). Figures 3D,E and Movie S11 in Supplementary Material illustrate the motile behavior of one resident CD8 T cell that migrates rapidly in a loose-collagen region and then reduces its velocity when encountering an obstacle composed of a bundle of dense collagen fibers. Notably, not all CD8 T cells were motile in loose-collagen areas, likely reflecting the presence of elements other than the ECM that limit T cells in their displacements.

As previously described in lung tumors, the fast moving CD8 T cells often exhibited straight migration paths. A close examination revealed that these cells follow precisely the pre-defined collagen scaffold and often migrate between two fibers, as if confined into a conduit (Figure 3F and Movie S12 in Supplementary Material). We then quantified the motile behavior of CD8 T cells in relation to collagen fiber linearity. In organized and linear matrix areas, CD8 T cells migrated in a relatively linear fashion, as based on straightness indices close to 0.6 on average (Figure 3G). By contrast, these indices were much smaller when CD8 T cells were tracked in non-organized collagen fibers. Overall, our results indicate that in human ovarian carcinomas a fibrous stroma can act both as a physical barrier and a guidance structure for resident CD8 T cells.

### Back and forth migration of resident CD8 T cells in the stroma of human ovarian carcinomas

**Figure 4:**
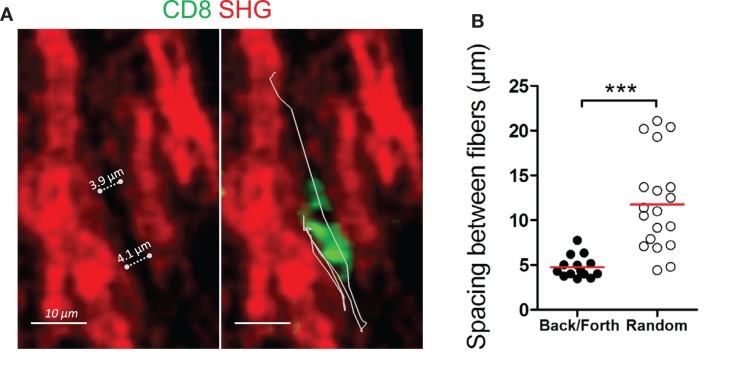
**Back and forth migration of resident CD8 T cells in the stroma of human ovarian carcinomas**. **(A)** Representative image of a resident CD8 T cell that exhibits a back and forth migration mode relative to collagen fibers. See also Movie S13 in Supplementary Material. Left: SHG signal (red) alone. Dotted lines indicate the spacing between collagen fibers. Right: same SHG image as in left but with a CD8 T cell (green) and its trajectory (white line) during a 17-min recording. **(B)** Spacing between collagen fibers are shown for CD8 T cells that display either back and forth or random migration. The durations of the tracks were non-significantly different within the two groups (13.5 ± 2.6 min for CD8 T cells that exhibit back and forth migration vs. 16.8 ± 2.6 min for CD8 T cells that migrate randomly). Values are compiled from four different ovarian tumors. *t*-test, *P < 0.05; **P < 0.01; ***P < 0.001.

In some ovarian tumor biopsies (four out of nine), we noted the presence of motile CD8 T cells that exhibited back and forth migration (Figure 4A and Movies S13 and 14 in Supplementary Material). Characteristic aspects of this behavior were phases of migration along a straight path, interrupted by short migration pauses during which CD8 T cells reoriented their locomotion machinery and migrated in the opposite direction. These alternate forward and backward movements can be repeated a number of times during a 20 min recording. Among motile CD8 T cells in these four ovarian tumors, 20–40% of the tracks displayed this type of locomotion. Notably, this mode of migration was rarely observed in lung tumors. We found that this type of cell movement was strongly dependent on the structure of the stromal collagen network and in particular on its porosity. Our data indicate that CD8 T cells with alternate forward and backward motion migrate in a conduit-like structure, with pore sizes ranging between 4 and 7 μm (Figure 4B). The available spaces between collagen fibers perfectly accommodate the moving T cell body. In other regions, the structure of the collagen network was very different, with CD8 T cells migrating randomly where the spacing between two fibers was significantly higher (11.79 ± 1.207 μm). These findings led us to postulate a mechanism by which space restriction constrains the movement of CD8 T cells in the tumor stroma and forces them to migrate with alternate forward and backward movements.

### The collagen structure surrounding tumor islets controls the capacity of CD8 T cells to contact tumor cells

**Figure 5:**
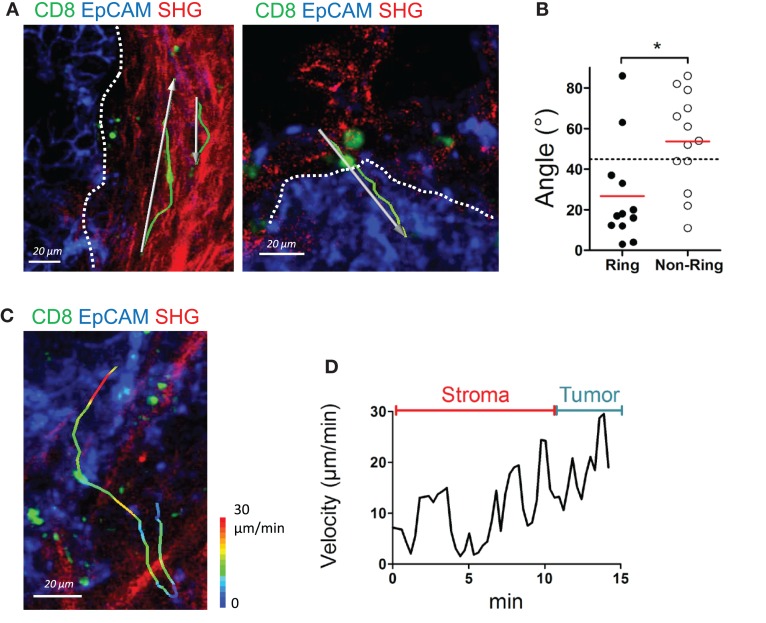
**The structure of the collagen around tumor islets dictates the migration of resident CD8 T cells**. **(A)** Representative images of resident CD8 T cells migrating in peritumoral areas surrounded by ring (left) and non-ring (right) structures. See also Movie S15 in Supplementary Material. Tracks (green lines) and trajectory vectors (white arrows) during a 20-min recording are also shown. Dotted lines denote tumor–stroma interface. **(B)** Angle between tumor–stroma boundary and trajectory vector of individual CD8 T cells during a 20-min recording, measured in peritumoral regions with ring or non-ring fibrillar structures. Dashed line denotes 45°, the expected result from a cell population migrating with no directional bias. Values were from four different ovarian tumor specimens. **(C)** Representative image of a CD8 T cell that migrates from the stroma to the tumor cell region in a specimen devoid of ring structure around tumor islets. The track of the CD8 T cells is color-coded according to the instantaneous velocity. See also Movie S16 in Supplementary Material. **(D)** Velocity of the CD8 T cell represented in **(C)**. Mann–Whitney test, *P < 0.05.

In human lung tumors, we previously demonstrated that the presence of a dense collagen network, composed of thick and linear ECM fibers surrounding tumor islets, limits T cell trafficking (11). From this study, we suggest that these concentric surrounding structures, that we have termed *ring structures*, observed in some but not all human lung carcinomas, contribute to keeping T cells in the stroma, away from tumor cells. One important question is whether such signature is a specific feature of lung carcinomas or is also observed in other solid malignancies. We thus examined the organization of the collagen network adjacent to tumor islets in ovarian carcinomas and their influence on resident CD8 T cells. Analysis performed on nine ovarian tumors showed the presence of such ring structures composed of dense and parallel collagen fibers surrounding tumor islets, in three tumors (Figure 5A left panel). In all the other tumors, the collagen network most proximal to tumor cells did not show a particular organization (Figure 5A right panel). In some tumors, the SHG signal was dim with small fibers oriented in no particular direction. In one tumor, strands of collagen were even found penetrating into the tumor mass. We then monitored the motility of T cells in these organized and non-organized peritumoral areas. Resident CD8 T cell motility tracked in organized peritumoral regions displayed trajectories mostly parallel to the tumor-stroma interface (Figure 5A left panel and Movie S15 in Supplementary Material). The situation was strikingly different in non-organized peritumoral regions, with T cell tracks that were randomly distributed (Figure 5A right panel). To quantify the CD8 T cell motility pattern in organized and in non-organized peritumoral areas, we analyzed the trajectory vectors of individual lymphocytes, i.e., the connecting lines between starting point and end point of each track (11). Measurement of the smallest angle between the vector and the tumor-stroma boundary resulted in values substantially smaller than 45° (26.8 ± 7.18°, *n* = 12 cells, Figure 5B) when CD8 T cells were in peritumoral ring structures. In contrast, widely distributed angles resulting in average values close to 45° (53.8 ± 6.52°, *n* = 13 cells), thus indicating a random migration, were observed when T cells migrated in non-organized peritumoral areas. The random migration of CD8 T cells in non-organized peritumoral areas prompted us to investigate the dynamics of lymphocytes navigating from the stroma to tumor islets. The migration of CD8 T cells through these distinct microenvironments is supposed to be important for an effective destruction of tumor cells. Analysis of velocities of the few CD8 T cells at the stroma-tumor boundary did not reveal marked speed changes when T cells crossed this boundary. Large speed fluctuations were observed when T cells migrated within the stroma and similar variations occurred once T cells reached tumor cell regions (Figures 5C,D and Movie S16 in Supplementary Material).

We conclude that the architecture of the collagen network surrounding tumor islets likely contribute to dictate the migration of resident CD8 T cells and their ability to contact tumor cells. In absence of ring structures, CD8 T cells seem to be able to migrate without hindrance from the stroma to tumor islets.

## Discussion

For an effective direct destruction of cancer cells, CD8 T cells must fulfill several functions. First, they should be able to migrate efficiently into and within tumors in order to make contact with malignant cells and antigen-presenting cells. Second, they should be able to respond adequately to tumor antigens by releasing cytotoxic granules. In cancer patients, accumulating evidence suggests that both T cell trafficking and responsiveness to tumor antigens are altered (2). Whereas a lot of efforts have been made to overcome T cell exhaustion, boosting lymphocyte activation alone would not be enough if T cells cannot migrate and reach tumor cells. It is therefore important to understand the cellular and molecular processes underlying T cell motility within tumors in order to optimize the current immunotherapeutic strategies aiming at increasing T cell cytotoxicity. Here, we have used and refined our previously described slice assay to examine the migration of resident CD8 T cells in progressing human ovarian and lung tumors. Our imaging study uncovered several novel findings.

First, we observed that in ovarian carcinomas CD8 T cells are much more numerous in the stromal compartments than in tumor islets. This result extends the observations that we had initially made in human lung carcinomas. It is in line with previous studies in which CD8 T cells have been shown to be similarly enriched in the stroma of a large number of human solid malignancies, including ovarian carcinomas (2, 14). Of interest, the number of CD8 T cells presents in tumor cell regions of ovarian tumors was correlated with a better outcome (14). Additionally, a recent study revealed that the exclusion of T cells from tumor islets predicts unresponsiveness to anti-PD-1 treatment in some cancer patients (17). Thus, the trapping of T cells into the stroma appears to be a general feature of growing solid tumors and may be associated with immunsuppression.

Our data also indicate that a large proportion of T cells slowly migrate in the stromal compartment. The slow migration of a large number of CD8 T cells is consistent with previous studies showing an altered migratory pattern of T cells during states of functional impairment. For instance, during persistent viral infection T cells were found to be in a state of motility paralysis (24). Likewise, by looking at the dynamics of endogenous T cells in progressively growing murine tumors, Mrass et al. reported a low T cell motility (4), a finding recently confirmed in two different murine tumor models (8, 9). In contrast, an increased T cell motility within tumors is associated with successful immunotherapy treatments (4, 9). Among the different reasons that contribute to explain the relative poor motility in growing tumors, a lack of migration cues including chemokines, TCR stimulation, and costimulatory molecules have been put forward (18, 25). One hallmark of progressive tumors is the deposition of a dense collagen network, which has been long recognized to support cancer cell growth and metastatic dissemination (26). Moreover, there is now compelling evidence demonstrating that dense collagen structures hinder antitumor immune responses, including T cell migration. In human lung tumors, our published study indicated that T cell positioning and migration was markedly reduced in matrix-rich stromal regions (11). Likewise, in pancreatic carcinomas, one of the most fibrotic tumors, T cells accumulate in areas of low-density collagen, far from tumor islets (15, 27). In addition, by comparing the recruitment of T cells into various murine metastatic melanomas following chemotherapy, Tan et al. made the interesting observation that T cell infiltration only occurred in tumors whose stroma exhibited a loose matrix architecture (28). In our study, we extended these findings to resident CD8 T cells in ovarian and lung carcinomas that are also characterized by an extensive collagen-rich stroma. Of note, most of these studies are correlative and at present we do not known whether an altered ECM is directly responsible for the poor capacity of T cells to migrate within the tumor stroma. An exaggerated ECM deposition is not only a hallmark of growing tumors, but also of fibrotic tissues. Interestingly, a negative role of a dense ECM on T cell immune surveillance was recently reported during liver fibrosis (29).

The tumor stroma is a complex and heterogeneous microenvironment composed of cells and elements that cooperate to influence, either positively or negatively, T cell migration (30). Along with very dense matrix areas, some stromal regions are also characterized by organized and linear collagen fibers that guide T cells in their displacements. Accordingly, a large proportion of motile CD8 T cells in the stroma exhibited straight migratory paths along collagen fibers. Notably, some linearly migrating lymphocytes also display alternate forward and backward movements, especially when positioned between two collagen fibers with gap sizes that accommodate the T cell body. This type of “back and forth” migration which, to our knowledge, has never been reported for lymphocytes, presents some analogies with that described for macrophages in dense collagen gels (31). In this setting, macrophages have been shown to generate a persistent tunnel, via proteases, which is further used by the following macrophages to migrate rapidly and often with oscillatory movements. T cells, known to migrate in a MMP-independent manner, might use similar tunnels previously generated by other cells using their proteases. Why T cells exhibit such oscillatory behavior when present in these “tubes” is unknown too. Even if we cannot rule out the presence of obstacles – dense ECM or unlabeled cells – that would force T cells to stop and reorient their locomotion machinery, we favor the existence of a constraining structure, in which chemokines are present (there is a marked T cell motility), but without gradient (there is no directionality). T cell motility parameters, such as speed and propensity to turn, have also been shown to be cell-intrinsic (32). For instance, when placed in large micro-channels, T cells did not show persistent migration along the channel, but instead switched from one wall to another (33). Two-photon imaging studies have also confirmed the fact that T cells exhibit, in a number of tissues, repetitive straight runs, followed by periods of pauses (34). This “stop and go” mode of migration has been recognized to facilitate the detection of rare expressed antigens by naïve T cells in lymphoid organs and the rapid search of target cells by effector T cells in inflamed tissues. In tumors, the prevalence of linear collagen structures along with dense collagen networks may constrain such T cell search strategy and thus compromise tumor immunosurveillance.

These ECM structures present around tumor islets will also be important in controlling the infiltration of T cells into tumor islets. By analyzing T cell dynamics in lung and ovarian tumors in which islets were surrounded or not by adjacent collagen strands, our data support the notion that a ring structure is detrimental for CD8 T cells to migrate from the stroma to tumor cell regions, in line with our previous study (11). In absence of such determinants, we observed some T cells that enter into tumor islets without particular deceleration. Apart from a key role played by the ECM, additional mechanisms might also be operative in preventing T cells from contacting tumor cells. A recent study has proposed that the paucity of T cells observed in tumor islets of pancreatic adenocarcinoma was due to the presence of CXCL12 in cancer cell regions, creating a chemical barrier against T cells (19). Depending on the tumor type, the crossing of the tumor-stroma boundary might be favored or hindered by a number of structural and molecular determinants including a chemokine gradient (either attractive or repulsive) and physical constraints.

Once passed these barriers, we have observed that the rare T cells in tumor islets migrate more actively in these compartments than in the stroma. Thus, tumor cell regions cannot be considered as unfavorable migration zones for CD8 T cells, at least for some of them. This process might show some analogies to the crossing of T cells through activated venular walls, where lymphocytes undergo profound shape changes to extravasate (35).

Altogether, our data support the notion that the structure of stromal ECM has overall a negative influence on resident CD8 T cells, limiting their migration in the stroma and preventing them from contacting tumor cells. These mechanisms may impede endogenous antitumor immune responses and should be considered in the development of novel immunotherapy strategies. Particularly relevant to this issue is the recent report of chimeric antigen receptor T cells engineered to express ECM-degrading enzymes that has shown good infiltration into tumors and antitumor efficacy (36).

The approach that we have developed offers a number of interesting perspectives in a context of human tumor immunology. T cells, as well as other cells for which antibodies have been generated, can be imaged with excellent specificity and their interplays studied in real time. For instance, the monitoring of particular subsets of T cells, including activated and exhausted lymphocytes, with antibodies directed against specific markers like PD-1 represents a promising strategy.

This experimental system also presents some limitations that need to be kept in mind. Damage associated with the slicing may affect T cell functioning, especially in the superficial region of the tissue near the cut surface. We have bypassed this problem by imaging cells located at several tens of microns from the surface, in presumably healthier regions of the tissue. Another potential concern is the use of full-sized antibodies that can affect the behavior of the tracked cells. We have no indication that our data were affected by such problems, since CD8 T cells exhibited a good motility that very much depends on the tumor environment and in particular on the structure of the ECM. We confirmed some of our results with Fab fragments of anti-CD8 that present several advantages compared to full-sized antibodies. Small molecules and devoid of a Fc region, Fab fragments probably penetrate more efficiently into tissues, without binding to cells that express Fc receptors. Although Fab fragments, like full-sized antibodies, keep their ability to modulate the interaction between CD8 and MHC class I molecules, they are poorly able to transmit an intracellular activation signal capable of altering the T cell behavior. The recent use of camelid-derived antibody fragments, much smaller than Fab fragments, represents an interesting alternative strategy to image the progress of an immune response in live tissues (37).

It is clearly established that the immunosuppressive environment of solid tumors limits the migration and functions of T lymphocytes and therefore their antitumor activities. Prior to initiating clinical trials, model systems in which novel immunotherapeutic molecules can be characterized and tested for their potency and safety should be available. So far, most of these assays are based on *in vitro* cell culture systems that poorly mimic the complexity of the tumor tissue. We believe that the approach we have developed – tracking of immunostained CD8 T cells in fresh human tumor tissue with imaging technology – could be used as pre-clinical model system in which novel immunotherapy treatments and especially those designed to boost T cell migration can be assessed and optimized in conditions close to the clinic.

## Author Contributions

EP, HB, and ED designed the study; EP and HB performed live imaging experiments; EP, HB, and ED analyzed imaging experiments; AM-L, MA, CN, DD, M-ALF-B provided human tumors; ED wrote the manuscript with important input from EP and HB.

## Conflict of Interest Statement

The authors declare that the research was conducted in the absence of any commercial or financial relationships that could be construed as a potential conflict of interest.

## Supplementary Material

The Supplementary Material for this article can be found online http://journal.frontiersin.org/article/10.3389/fimmu.2015.00500
