# Supplementary material for: Real-Time Imaging of Resident T Cells in Human Lung and Ovarian Carcinomas Reveals How Different Tumor Microenvironments Control T Lymphocyte Migration
Source: Front Immunol. 2015 Oct 12;6:500. doi: 10.3389/fimmu.2015.00500 (PMC4600956; doi:10.3389/fimmu.2015.00500)
Supplement: Supplementary file 1 [file Presentation_1.ZIP › Movies/Movie Captions.pdf]

Supplementary material

Movie 1: Resident CD8 T cell migration imaged using two-photon microscopy in an ovarian carcinoma slice. The slice stained with anti-CD8 (green) and anti-EpCAM (blue) antibodies was imaged during 13 min with a two-photon microscope. Frame interval is 20 s. The SHG signal is in red. The animation represents a three-dimensional (3D) reconstruction of a sequential *z* series. Note the rapid bleaching of the fluorescence.

Movie 2: Resident CD8 T cell migration imaged using a combination of confocal and two-photon microscopy in an ovarian carcinoma slice. The slice stained with anti-CD8 (green) and anti-EpCAM (blue) antibodies was imaged during 25 min with a confocal microscope. Frame interval is 20 s. The SHG signal (red) was recorded at the end of the time-lapse with a two-photon microscope. The animation represents a three-dimensional (3D) reconstruction of a sequential *z* series. Note the absence of bleaching of the fluorescence.

Movie 3: Resident CD8 T cells hardly migrate at the cut surface of the slice. The ovarian tumor slice stained with anti-CD8 (green) and anti-EpCAM (blue) antibodies was imaged during 14 min with a confocal microscope. Frame interval is 20 s. The animation represents a single section of 10 µm, taken 3 µm below the cut surface of the slice.

Movie 4: Some resident CD8 T cells actively migrate deep within the slice. The ovarian tumor slice stained with anti-CD8 (green) and anti-EpCAM (blue) antibodies was imaged during 14 min with a confocal microscope. Frame interval is 20 s. The animation represents a single section of 10 µm, taken 32 µm below the cut surface of the slice.

Movie 5: Resident CD8 T cells slowly migrate in the stroma of ovarian tumors. The slice stained with anti-CD8 (green) and anti-EpCAM (blue) antibodies was imaged during 30 min with a confocal microscope. Tracks are color coded according to CD8 T cell displacement length. The SHG signal (red) was recorded at the end with a two-photon microscope. Frame interval is 20 s. The animation represents a three-dimensional (3D) reconstruction of a sequential *z* series. A still image is shown in Fig. 2C.

Movie 6: Resident CD8 T cells migrate actively in tumor islets of ovarian tumors. The slice stained with anti-CD8 (green) and anti-EpCAM (blue) antibodies was imaged during 30 min with a confocal microscope. The SHG signal (red) was recorded at the end with a two-photon microscope. Frame interval is 20 s. The animation represents a three-dimensional (3D) reconstruction of a sequential *z* series.

Movie 7: Resident CD8 T cells migrate actively in tumor islets of ovarian tumors. The slice stained with anti-CD8 (green) and anti-EpCAM (blue) antibodies was imaged during 30 min with a confocal microscope. Frame interval is 20 s. The animation represents individual z planes captured at 13 µm (left panel) and 41 µm (right panel) from the cut surface.

Movie 8: Resident CD8 T cells migrate actively in tumor islets of lung tumors. The slice stained with anti-CD8 (green) and anti-EpCAM (blue) antibodies was imaged during 20 min with a confocal microscope. Frame interval is 20 s. The animation represents individual z planes captured at 15 µm (left panel) and 33 µm (right panel) from the cut surface.

Movie 9: Resident CD8 T cell migration assessed with a fluorescently labeled Fab fragment directed against CD8. The slice stained with anti-CD8 Fab fragments (green) antibodies was imaged during 13 min with a confocal microscope. The SHG signal (red) was recorded at the end with a two-photon microscope. Frame interval is 20 s. The animation represents a three-dimensional (3D) reconstruction of a sequential *z* series. Note that a large fraction of CD8 T cells are sessile in the stroma with only a few that actively migrate.

Movie 10: Resident CD8 T cells that migrate actively in loose collagen regions of an ovarian carcinoma slice. The slice stained with anti-CD8 antibodies (green) and anti-EpCAM antibodies (blue) was imaged during 14 min with a confocal microscope. The SHG signal (red) was recorded at the end with a two-photon microscope. Tracks are color coded according to CD8 T cell displacement length. Frame interval is 20 s. The animation represents a three-dimensional (3D) reconstruction of a sequential *z* series.

Movie 11: Example of a CD8 T cell that rapidly migrates in loose collagen area and then reduces its velocity when encountering a bundle of dense collagen fibers. The slice stained with anti-CD8 antibody (green) was imaged during 30 min with a confocal microscope. The SHG signal (red) was recorded at the end with a two-photon microscope. Tracks are color coded according to CD8 T cell instantaneous velocity. Frame interval is 20 s. The animation represents a single section of 10 µm. Snapshots are shown in Fig. 3D.

Movie 12: Example of a CD8 T cell that presents a straight migration path along linear collagen fibers. The slice stained with anti-CD8 antibody (green) was imaged with a confocal microscope. The SHG signal (red) was recorded at the end with a two-photon microscope. Tracks are color coded according to CD8 T cell instantaneous velocity. Frame interval is 20 s. The animation represents a single section of 10 µm. Snapshots are shown in Fig. 3F.

Movie 13: Example of a CD8 T cell that presents a back and forth mode of migration. The slice stained with anti-CD8 antibody (green) was imaged during 17 min with a confocal microscope. The SHG signal (red) was recorded at the end with a two-photon microscope. The cell trajectory is in white. Frame interval is 20 s. The animation represents a single section of 10 µm. A still image is shown in Fig. 4A.

Movie 14: Another example of a CD8 T cell that presents a back and forth mode of migration. The slice stained with anti-CD8 antibodies (green) and anti-EpCAM antibodies (blue) was imaged during 16 min with a confocal microscope. The SHG signal (red) was recorded at the end with a two-photon microscope. The cell trajectory is in white. Frame interval is 20 s. The animation represents a single section of 10 µm.

Movie 15: CD8 T cells in organized peritumoral areas exhibited straight migration paths along collagen fibers, parallel to the tumor-stroma boundary. The slice stained with anti-CD8 (green) and anti-EpCAM antibodies (blue) was imaged during 20 min with a confocal microscope. The SHG signal (red) was recorded at the end with a two-photon microscope. CD8 T cell trajectories are in green. Frame interval is 20 s. The animation represents a three-dimensional (3D) reconstruction of a sequential *z* series. A still image is shown in Fig. 5A.

Movie 16: Example of a CD8 T cell that migrate from the stroma to tumor cell regions without changes in its velocity. The slice stained with anti-CD8 (green) and anti-EpCAM antibodies (blue) was imaged during 18 min with a confocal microscope. The SHG signal

(red) was recorded at the end with a two-photon microscope. The track is color coded according to the cell instantaneous velocity. Frame interval is 20 s. The animation represents a three-dimensional (3D) reconstruction of a sequential *z* series.
